# Clearance of a persistent picornavirus infection is associated with enhanced pro-apoptotic and cellular immune responses

**DOI:** 10.1038/s41598-017-18112-4

**Published:** 2017-12-19

**Authors:** Carolina Stenfeldt, Michael Eschbaumer, George R. Smoliga, Luis L. Rodriguez, James Zhu, Jonathan Arzt

**Affiliations:** 1 Foreign Animal Disease Research Unit, USDA-ARS, Plum Island Animal Disease Center, Greenport, NY USA; 2 Department of Veterinary population medicine, University of Minnesota, St. Paul, MN USA; 3 Institute of Diagnostic Virology, Friedrich-Loeffler-Institut, Greifswald, Insel Riems Germany

## Abstract

Long-term persistent viral infections cause substantial morbidity and associated economic losses in human and veterinary contexts. Yet, the mechanisms associated with establishment of persistent infections are poorly elucidated. We investigated immunomodulatory mechanisms associated with clearance versus persistence of foot-and-mouth disease virus (FMDV) in micro-dissected compartments of the bovine nasopharynx by microarray. The use of laser-capture microdissection allowed elucidation of differential gene regulation within distinct anatomic compartments critical to FMDV infection. Analysis of samples from transitional and persistent phases of infection demonstrated significant differences in transcriptome profiles of animals that cleared infection versus those that became persistently infected carriers. Specifically, it was demonstrated that clearance of FMDV from the nasopharyngeal mucosa was associated with upregulation of targets associated with activation of T cell-mediated immunity. Contrastingly, gene regulation in FMDV carriers suggested inhibition of T cell activation and promotion of Th2 polarization. These findings were corroborated by immunofluorescence microscopy which demonstrated relative abundance of CD8^+^ T cells in the nasopharyngeal mucosa in association with clearance of FMDV. The findings presented herein emphasize that a critical balance between Th1 and Th2 -mediated immunity is essential for successful clearance of FMDV infection and should be considered for development of next-generation vaccines and antiviral products.

## Introduction

Foot-and-mouth disease virus (FMDV), the prototype *Aphthovirus* within the *Picornaviridae* family, is the causal agent of foot-and-mouth disease (FMD), a highly contagious disease of cloven-hoofed animals^1^. The endemic presence of FMD in large regions of the world compromises food security and animal welfare. Additionally, as a country’s official FMD status defines access to international markets for export of animal products, this disease has a substantial global economic impact on agricultural production and trade.

Control and eradication of FMD is impeded by the existence of a prolonged subclinical persistent phase of infection in ruminant species^2–4^. This concept, generally referred to as the FMDV carrier state, is specifically problematic when vaccination is used to control FMD in non-endemic regions as vaccinated cattle may maintain subclinical FMDV persistence following virus exposure, despite having been protected against the clinical disease^5–12^. Confirmed evidence of FMDV transmission from persistently infected cattle is lacking. However, because infectious FMDV is detectable in oropharyngeal fluid (OPF) and nasopharyngeal tissues of carriers for prolonged periods of time, the presence of such animals precludes achieving an official status as FMD-free by the international organization for animal health (OIE)^13^.

In cattle, persistent FMDV has been localized to the follicle-associated epithelium (FAE) of the nasopharyngeal mucosa^10,11^ or associated lymph nodes^14^. FMDV is capable of persisting at these apparently privileged sites, despite induction of a strong systemic immune response^15–17^. Contrastingly, there is no microscopically identifiable local inflammatory response or pathological changes within nasopharyngeal tissues associated with FMDV persistence^11^.

Recent works from our laboratory have indicated inhibition of the local anti-viral response during FMDV persistence by associating down-regulated expression of a select panel of anti-viral host factors with detection of FMDV RNA in micro-dissected samples of nasopharyngeal epithelium^11^. Specifically, there were significant negative correlations in FMDV carriers between the quantity of FMDV RNA and the relative expression levels of interferon (IFN)-λ, IFN-γ, IFN regulatory factor (IRF)-7, and CXCL10 in micro-dissected samples of nasopharyngeal FAE^11^. This finding distinctly contrasts the significant induction of inflammatory cytokines and anti-viral host factors that occurs during acute FMDV infection in cattle^18–20^. Additionally, an extensive analysis of gene expression in nasopharyngeal tissue samples using whole tissue macerates from FMDV carriers and a bovine whole transcriptome microarray suggested that the FMDV carrier state was associated with impairment of apoptotic pathways and overexpression of genes associated with induction of regulatory T cells and T cell exhaustion^21^. A previous investigation has suggested that regulation of the early immune response to FMDV infection by high systemic levels of IL-10 may predispose to establishment of persistent infection^22^. However, although acute FMDV infection in pigs is associated with substantial induction of systemic IL-10 ^23^, pigs efficiently clear FMDV infection and there is no FMDV carrier state in suids^24^.

This current investigation further expands the effort to identify immunological mechanisms involved in the divergence between FMDV carriers and cattle that clear infection. Isolation of distinct microanatomic regions of the highly heterogeneous bovine nasopharynx, by use of laser-capture microdissection (LCM), enabled precise characterization of transcriptome profiles associated with defined stages of FMDV infection. This was based upon previous works which had enabled determination of the temporal window during which FMDV is cleared from the nasopharyngeal mucosa in animals that do not develop persistent infection^11^.

The work presented herein suggests a significant association between induction of a cytotoxic cellular immune response and efficient clearance of FMDV infection from the bovine nasopharyngeal mucosa. Additionally, upregulation of targets associated with induction of apoptotic or anti-proliferative pathways was associated with virus clearance whereas markers of an enhanced humoral immune response were found in samples from persistently infected FMDV carriers. The findings of the current investigation expand previous knowledge and provide additional novel insights into the mechanisms of FMDV persistence.

## Results

### Categorization of animal cohorts

This current study was based upon analyses of tissue samples harvested from animals that were part of a large scale experimental investigation of the FMDV carrier state divergence. Detailed descriptions of the overarching study design and characterization of disease progression have been previously published^11^. In brief; the temporal window of the divergence between cattle that maintained persistent infection (“carriers”) and those that successfully cleared infection (“non-carriers”) was determined through monitoring of FMDV shedding in oropharyngeal fluid (OPF) in a large cohort of animals (n = 46) experimentally infected with FMDV A_24_ Cruzeiro^11^. This led to the definition of the transitional phase of infection as 8–21 days post infection (dpi), which corresponded to the phase during which cattle were in the process of clearing infection or transitioning to become persistently infected carriers. Animals that were euthanized for tissue harvest during the transitional phase of infection were categorized as FMDV-positive or negative (“transitional carriers” or “transitional terminators”) based on isolation of FMDV from OPF and tissues at the time of euthanasia. By a similar approach, animals that were euthanized during persistent infection (post 28 dpi) were characterized as either FMDV carriers or non-carriers. This current investigation included micro-dissected samples of follicle-associated nasopharyngeal epithelium (FAE) from 16 animals. Amongst these, six cattle had been euthanized during the transitional phase of infection (3 transitional carriers and 3 transitional terminators), and seven were euthanized during the persistent phase of infection (3 carriers and 4 non-carriers). Additionally, samples from three additional, un-infected animals, were included to determine baseline gene expression levels.

### Transcriptomic profiling of the FMDV carrier state divergence

**Figure 1:**
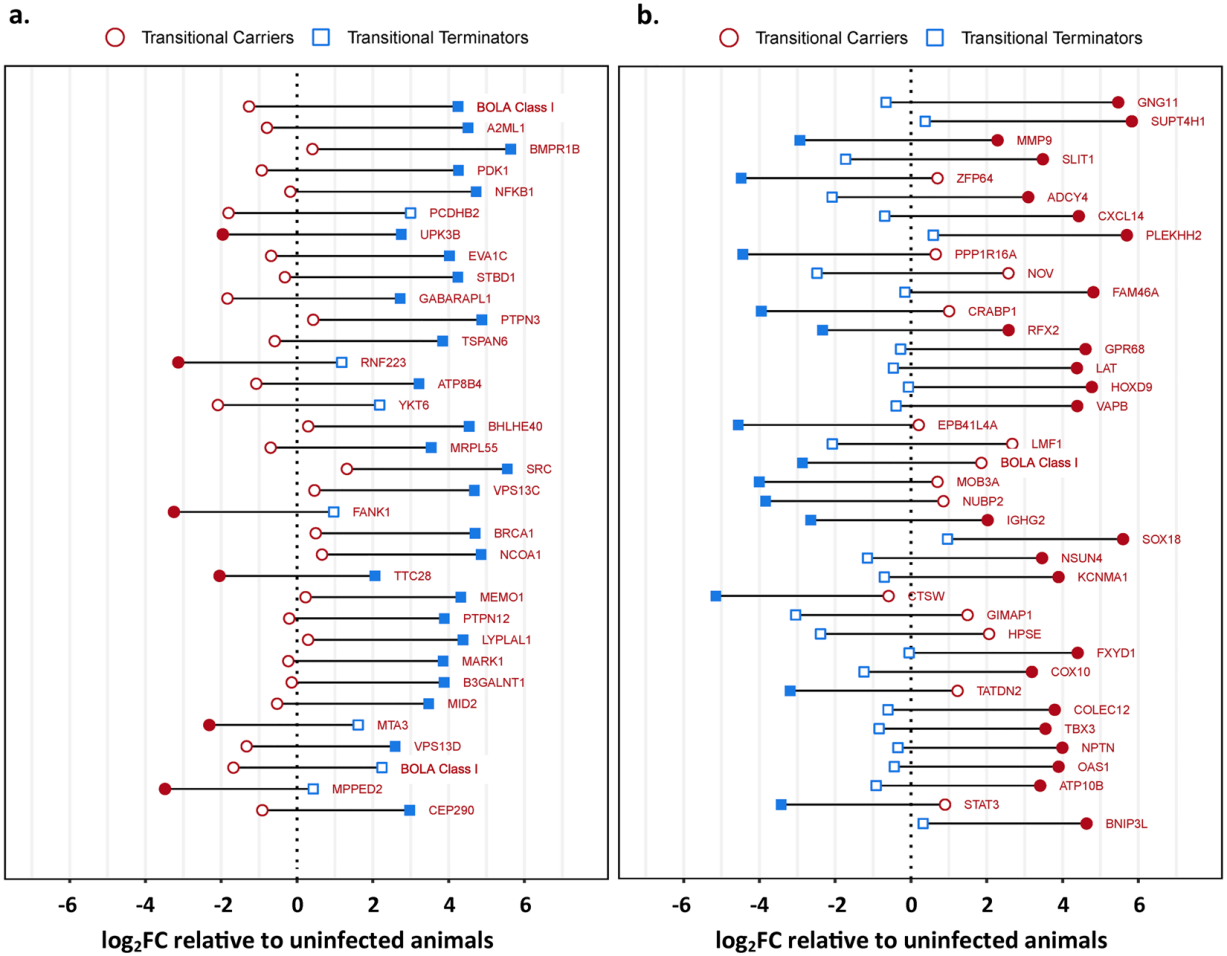
Differential gene expression between transitional carriers, transitional terminators and uninfected controls quantitated by microarray. The most strongly up- or downregulated probes (the top and bottom 0.1% log_2_FC) in the comparison between transitional terminators (blue squares) and transitional carriers (red circles) are shown ordered by decreasing difference. Genes that were expressed higher in transitional terminators are shown in panel a, and genes that were expressed higher in transitional carriers are shown in panel b. For each probe, the fold change relative to the uninfected controls is shown on the x-axis with the vertical dashed line representing no change compared to the uninfected animals. The horizontal distance between each blue square and red circle represents the difference in signal intensity between transitional terminators and transitional carriers. Filled blue (transitional terminators) or red (transitional carriers) symbols indicate a significant difference in intensity (adjusted p-value < 0.05) compared to the uninfected animals. The difference between transitional terminators and transitional carriers is significant (adjusted p-value < 0.05) for all probes shown.

**Figure 2:**
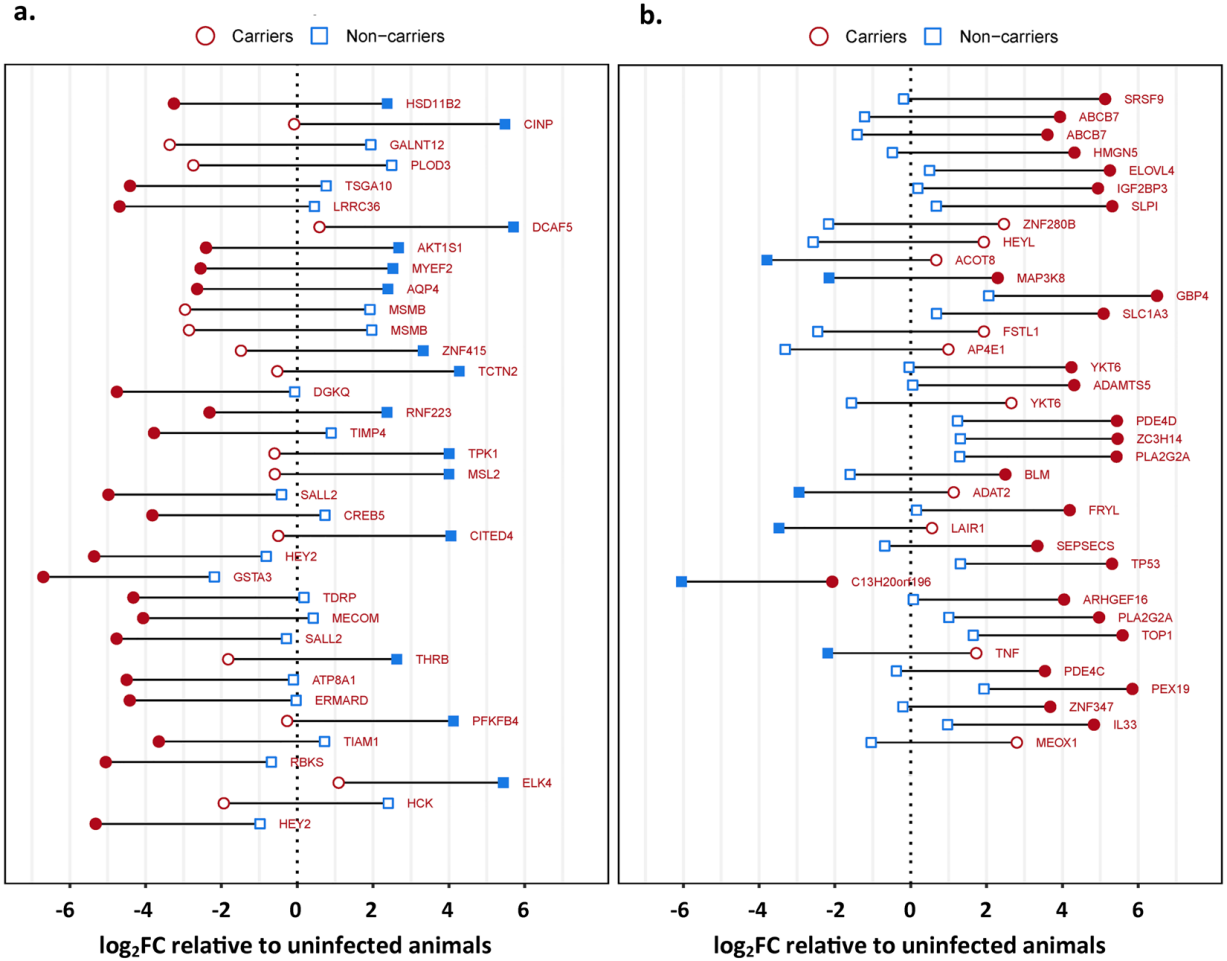
Differential gene expression between carriers, non-carriers and uninfected controls by microarray. The most strongly up- or downregulated probes (the top and bottom 0.1% log_2_FC) in the comparison between non-carriers (blue squares) and carriers (red circles) are shown ordered by decreasing difference. Genes that were expressed higher in non-carriers are shown in panel (a), and genes that were expressed higher in carriers are shown in panel (b). For each probe, the fold change relative to the uninfected controls is shown on the x-axis with the vertical dashed line representing no change compared to the uninfected animals. The horizontal distance between each blue square and red circle represents the difference in signal intensity between non-carriers and carriers. Filled blue (non-carriers) or red (carriers) symbols indicate a significant difference in intensity (adjusted p-value < 0.05) compared to the uninfected animals. The difference between non-carriers and carriers is significant (adjusted p-value < 0.05) for all probes shown.

For the objective of examining transcriptomic alterations associated with clearance versus persistence of FMDV, a bovine whole transcriptome microarray was used to quantify and compare expression levels of over 40 000 bovine mRNAs within and between animal cohorts using FAE samples generated by laser-capture microdissection (LCM). Gene expression levels, measured as microarray probe signal intensities within the four groups were compared to baseline expression levels of uninfected control animals. Additional pairwise comparisons were performed to quantify differences in gene expression between transitional carriers and transitional terminators, as well as between carriers and non-carriers. Differences in gene expression between groups were quantitated as the ratio of signal intensities which are presented as log_2_ fold-changes (log_2_FC). For pairwise comparisons, negative log_2_FC values indicate higher expression in transitional carriers or carriers, whereas positive log_2_FC values indicate higher expression in transitional terminators or non-carriers. The targets with the highest significant relative differences in expression between cohorts, corresponding to the top and bottom 0.1% of log_2_FC values with p-values ≤ 0.05, are presented in Figs 1 and 2. Within this filtered data set, there was an overrepresentation of genes from two overarching functional categories: regulation of apoptosis or cellular proliferation, or regulation of immune function. For each of the paired comparisons, differentially expressed genes grouped within either of these two functional categories are presented in Tables 1–4.

**Table 1:** Microarray analysis, Transitional phase: Differentially regulated genes associated with regulation of immune functions.

log_2_FC	Gene Symbol	Description	Average expression	Adjusted p-value
***Higher in Transitional Terminators***				
5.5	BOLA	Major histocompatibility complex class I alpha chain	10.0	1.49E-03
5.3	A2ML1	α-2 macroglobulin like protease inhibitor -1	10.5	9.38E-03
5.2	PDK1	Phosphoinositide-dependent protein kinase-1	7.3	7.05E-04
4.9	NFKB1	Nuclear factor of kappa light polypeptide gene enhancer in B-cells 1	7.4	3.19E-05
4.4	TSPAN6	Tetraspanin 6	8.2	4.09E-02
4.2	BHLHE40	Basic helix-loop-helix family member e40	10.5	2.72E-02
4.2	SRC	SRC proto-oncogene, non-receptor tyrosine kinase	8.5	1.70E-03
4.0	MID2	Midline 2	7.1	3.82E-03
3.9	BOLA	Non-classical major histocompatibility complex class I	11.8	4.32E-02
***Higher in Transitional Carriers***				
−4.3	STAT3	Signal transducer and activator of transcription 3	11.7	3.82E-03
−4.3	OAS1	2′,5′-oligoadenylate synthetase 1, 40/46 kDa	7.6	5.21E-04
−4.5	HPSE	Heparanase	9.5	3.45E-02
−4.5	GIMAP1	GTPase, IMAP family member 1-like (LOC512867)	7.7	2.57E-02
−4.6	CTSW	Cathepsin W	10.0	1.83E-03
−4.7	IGHG2	Immunoglobulin gamma 2a heavy chain constant region	14.3	6.15E-05
−4.7	BOLA	Major histocompatibility complex class I	11.5	2.46E-03
−4.8	LAT	Linker for activation of T cells	7.9	2.84E-04
−4.9	GPR68	G protein-coupled receptor 68, OGR1	8.6	3.88E-04
−4.9	RFX2	Regulatory factor X2	11.1	9.11E-04
−5.1	CXCL14	Chemokine (C-X-C motif) ligand 14	11.4	4.26E-02
−5.2	ADCY4	Adenylate cyclase 4	10.0	8.22E-03
−5.2	ZFP64	Zinc finger protein 64 homolog (mouse)	11.0	2.10E-02
−5.2	SLIT1	Slit homolog 1 (Drosophila)	9.8	1.32E-03
−6.1	GNG11	Guanine nucleotide binding protein (G protein), gamma 11	7.4	1.01E-03

**Table 2:** Microarray analysis, Transitional phase: Differentially regulated genes associated with regulation of cellular proliferation or apoptosis.

log_2_FC	Gene Symbol	Description	Average expression	Adjusted p-value
***Higher in Transitional Terminators***				
5.2	BMPR1B	Bone morphogenetic protein receptor, type IB	8.6	4.79E-02
4.4	PTPN3	Protein tyrosine phosphatase, non-receptor type 3	11.5	3.92E-03
4.3	ATP8B4	ATPase phospholipid transporting 8B4 (putative)	7.5	4.22E-03
4.2	FANK1	Fibronectin type III and ankyrin repeat domains 1	8.6	2.01E-02
4.2	BRCA1	Breast cancer 1, early onset	7.3	2.86E-02
4.1	PTPN12	Protein tyrosine phosphatase, non-receptor type 12	7.4	1.77E-02
3.9	MTA3	Metastasis associated 1 family, member 3	10.1	9.33E-03
3.9	MPPED2	Metallophosphoesterase domain containing 2	7.0	2.80E-03
***Higher in Transitional Carriers***				
−4.3	BNIP3L	BCL2/adenovirus E1B 19 kDa interacting protein 3-like	10.1	3.41E-02
−4.3	ATP10B	ATPase phospholipid transporting 10B (putative)	9.5	3.81E-02
−4.4	TBX3	T-box 3	8.2	2.10E-03
−4.6	CDKN2B-AS	Cyclin dependent kinase inhibitor 2B, antisense	10.5	4.79E-02
−4.6	SOX18	SRY (sex determining region Y)-box 18	9.1	1.44E-02
−4.8	HOXD9	Homeobox D9	6.2	1.56E-10
−5.0	CRABP1	Cellular retinoic acid binding protein 1	10.2	7.18E-04
−5.1	PPP1R16A	Protein phosphatase 1, regulatory (inhibitor) subunit 16A	10.2	1.01E-02
−5.1	NOV	Nephroblastoma overexpressed gene	10.7	6.47E-03
−5.2	MMP9	Matrix metalloproteinase 9 (type IV collagenase)	9.8	1.81E-04

**Table 3:** Microarray analysis, Persistent phase: Differentially regulated genes associated with regulation of immune functions.

log_2_FC	Gene Symbol	Description	Average expression	Adjusted p-value
***Higher in Non-Carriers***				
4.5	CREB5	cAMP responsive element binding protein 5	8.1	4.14E-03
4.5	CITED4	Cbp/p300-interacting transactivator, with Glu/Asp-rich carboxy-terminal domain, 4	10.4	1.10E-03
4.4	TIAM1	T-cell lymphoma invasion and metastasis 1	11.9	9.66E-05
4.3	ELK4	ELK4, ETS-domain protein (SRF accessory protein 1)	9.6	2.88E-02
4.3	HCK	Hematopoetic cell kinase, Src family tyrosine kinase	10.2	2.85E-02
***Higher in Carriers***				
−3.9	IL33	Interleukin 33	9.2	1.69E-02
−3.9	TNF	Tumor necrosis factor alpha	9.0	1.03E-03
−4.0	PLA2G2A	Phospholipase A2, group IIA	7.7	6.33E-03
−4.0	LAIR1	Leukocyte associated immunoglobulin like receptor 1	8.5	3.14E-02
−4.1	PLA2G2A	Phospholipase A2, group IIA	7.3	5.23E-03
−4.2	PDE4D	Phosphodiesterase 4D	7.0	3.72E-02
−4.3	ADAMTS5	ADAM metallopeptidase with thrombospondin type 1 motif, 5	6.4	1.55E-03
−4.4	GBP4	Guanylate binding protein 4	9.8	1.19E-02
−4.5	MAP3K8	Mitogen-activated protein kinase kinase kinase 8	8.9	1.67E-04
−4.6	SLPI	Secretory leukocyte peptidase inhibitor	7.0	5.47E-03

**Table 4:** Microarray analysis, Persistent phase: Differentially regulated genes associated with regulation of cellular proliferation or apoptosis.

log_2_FC	Gene Symbol	Description	Average expression	Adjusted p-value
***Higher in Non-Carriers***				
5.6	CINP	Cyclin-dependent kinase 2-interacting protein	10.3	7.38E-04
5.2	TSGA10	Testis specific, 10	9.2	7.98E-06
5.1	AKT1S1	AKT1 substrate 1 (proline-rich)	10.4	4.70E-04
4.6	SALL2	Sal-like 2 (Drosophila)	9.3	2.29E-03
4.5	SALL2	Sal-like 2 (Drosophila)	9.9	3.23E-03
4.4	ATP8A1	ATPase, aminophospholipid transporter, class I, type 8A, member 1	9.7	3.22E-03
***Higher in Carriers***				
−3.8	MEOX1	Mesenchyme homeobox 1	8.3	2.66E-02
−4.0	ARHGEF16	Rho guanine nucleotide exchange factor 16	7.4	1.02E-03
−4.0	TP53	Tumor protein p53	9.1	3.47E-02
−4.2	YKT6	YKT6 v-SNARE homolog (S. cerevisiae)	11.0	8.83E-03
−4.3	YKT6	YKT6 v-SNARE homolog (S. cerevisiae)	10.3	3.78E-02
−4.5	HEYL	Hairy/enhancer-of-split related with YRPW motif-like	11.7	1.54E-02
−4.6	ZNF280B	Zinc finger protein 280B	7.1	2.15E-03
−4.8	IGF2BP3	Insulin-like growth factor 2 mRNA binding protein 3	7.7	2.03E-08
−4.8	HMGN5	High mobility group nucleosome binding domain 5	7.7	5.17E-04
−5.3	SRSF9	Serine/arginine-rich splicing factor 9	8.3	3.06E-05

### Transitional phase, immune-regulatory

**Figure 3:**
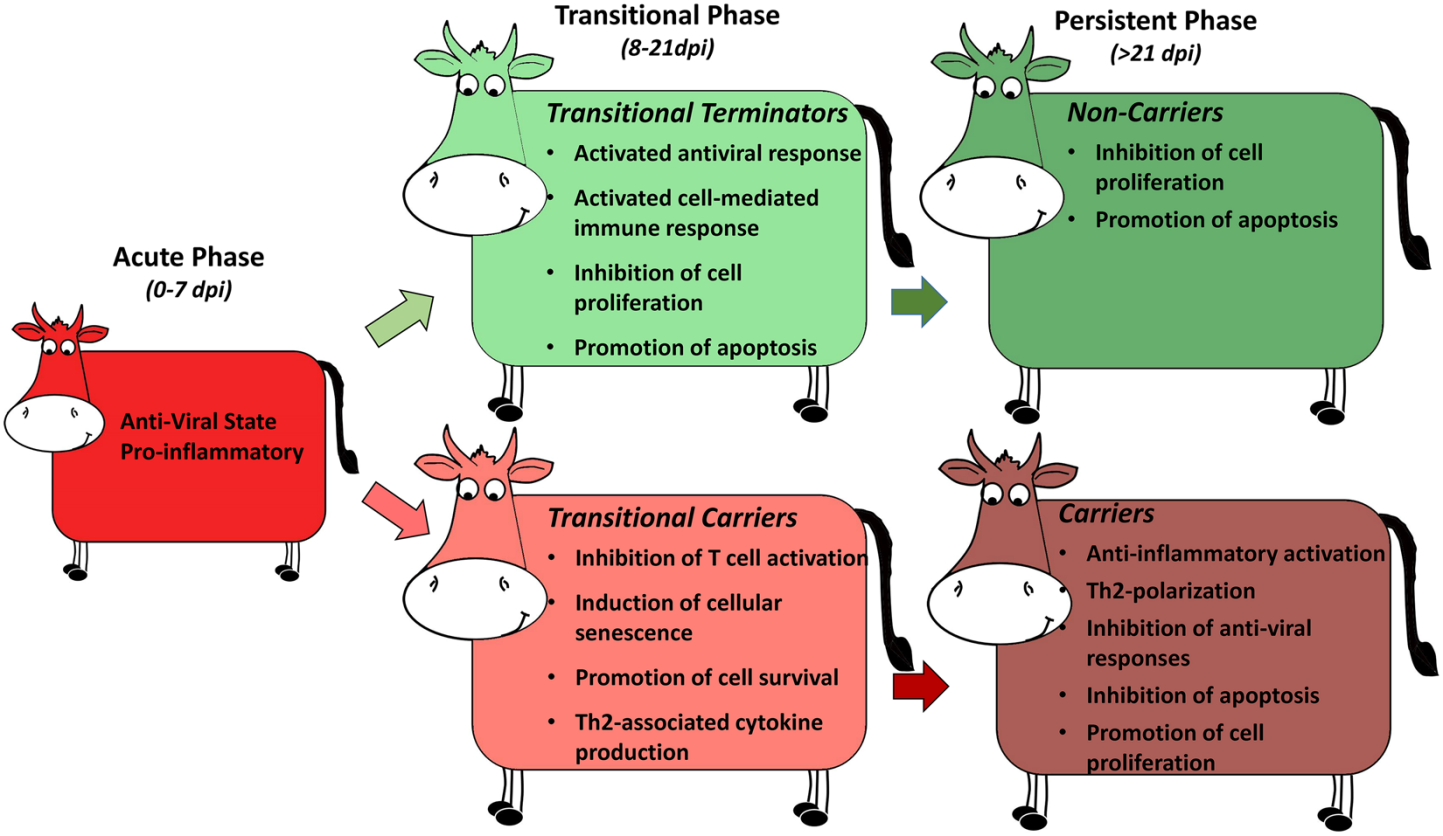
Overview of phases of FMDV infection indicating predominant trends of differential regulation of the host response. The transitional phase of FMDV infection bridges the acute and persistent phases, and constitutes the temporal window during which cattle either effectively clear infection, or develop into persistently infected FMDV carriers. In transitional terminators this phase is characterized by an activated antiviral response via induced cell-mediated immunity as well as induction of apoptosis-associated pathways. Contrastingly, the host response of the transitional carriers is dominated by inhibition of T cell activation and progression towards a Th2 polarization. The FMDV carrier state is characterized by a Th2-biased host response with sustained antibody-mediated immunity, downregulation of apoptotic pathways and activation of anti-inflammatory mechanisms.

Within the FAE of the transitional terminator cohort, there was an overrepresentation of gene targets that were associated with activation of a cellular or anti-viral response in the subset of overexpressed genes that were categorized as immune-regulatory (Table 1; Fig. 1a, Fig. 3). BOLA class I, the most up-regulated target in transitional terminators is one of the proteins most intrinsically associated with induction of cell mediated immunity^25^. Also among the most highly overexpressed targets, midline 2 (MID2, also known as TRIM1) is a ubiquitin ligase that is critically involved in cytotoxic granule exocytosis in CD8 T cells by regulation of the microtubule framework^26^. Additional overexpressed targets included basic helix-loop-helix family member e40 (BHLHE40) which is a transcription factor that is induced by IL-1β. Cellular BHLHE40 expression has been associated with production of IFN-γ, IL-17a, and granulocyte-macrophage colony-stimulating factor (CSF2) in T helper cells^27^ and invariant natural killer cells^28^. Similarly overexpressed, SRC family kinases are non-receptor kinases that mediate intracellular signaling events that are initiated upon ligation of antigen receptors on T cells and B cells^29,30^. Alpha-2 macroglobulin like protease inhibitor-1 (A2ML1) was the gene with the second highest relative expression in transitional terminators. This protein is an inhibitor of several classes of proteases, including chymotrypsin and papain, and it is generally expressed by keratinocytes^31^. Overexpression of this protein has been associated with resistance to HIV-1 infection^32^, and A2ML1 is thought to play a role in maintaining tissue integrity by inhibiting the action of extracellular proteases^31^.

Contrastingly, immune-regulatory targets that were highly overexpressed in transitional carriers were more likely to be associated with induction of cellular senescence or inhibition of cellular migration and T-cell activation (Table 1 Fig. 1b). The gene with the highest overexpression in transitional carriers was guanine nucleotide binding protein (G protein), γ-11 (GNG11). This protein is involved in transmission of extracellular stimuli to intracellular effectors, and upregulation of GNG11 has been strongly associated with induction of cellular senescence by activation of the extracellular signal-regulated kinase (ERK)1/2 pathway^33^. Similarly, the Slit homolog (SLIT1) was overexpressed in transitional carriers. This protein has been shown to interact with chemokine receptor CXCR4, thereby inhibiting leukocyte chemotaxis and interfering with T cell activation^34,35^. The inhibition of CXCR4 activity was further supported by the concurrent overexpression of CXCL14, which has been shown to bind and block the effects of CXCR4^36^ similar to SLIT1. Also overexpressed, the linker for activation of T cells (LAT) has been functionally associated with T cell development, although most specifically with development of regulatory T cells (Tregs) and thereby suppression of cellular immunity^37^. Overexpression of the zinc finger protein 64 homolog (ZFP64) has been associated with stimulation of TLRs, and leads to downstream production of IL-6, TNF-α, and IFN-β^38^. Regulation of IFN-β and TNF-α have previously been associated with acute and persistent phases of FMDV infection^20,39^ whereas IL-6 is a strong promoter of Th2 polarization^40,41^. The G protein-coupled receptor 68 (GPR68, also known as ovarian cancer G-protein coupled receptor; OGR1) was similarly overexpressed in transitional carriers. This receptor has also been shown to stimulate production of IL-6^42^, and can thereby also be linked to a Th2 polarization. Similarly, overexpression of the immunoglobulin gamma 2a heavy chain constant region (IGHG2), although a broad indicator, suggests an activated antibody-mediated immune response.

### Transitional phase, apoptosis and proliferation

Within the subset of strongly regulated genes that were associated with regulation of apoptosis or cellular proliferation, targets associated with induction of apoptosis or inhibition of cellular proliferation were overrepresented within the transitional terminators (Table 2; Fig. 1a). The gene within this functional category with the highest relative expression in transitional terminators was bone morphogenetic protein receptor 1B (BMPR1B). High expression of this protein has been associated with a direct inhibition of proliferation of keratinocytes^43^, as well as of human breast cancer cells^44^. Other targets that were overexpressed in transitional terminators and that have been directly associated with tumor suppression pathways included protein tyrosine phosphatase, non-receptor type 3 (PPTN3)^45^, fibronectin type III and ankyrin repeat domains 1 (FANK1)^46^, breast cancer 1, early onset protein (BRCA1)^47^, protein tyrosine phosphatase, non-receptor type 12 (PTPN12)^48^, metastasis-associated protein 3 (MTA3)^49^, and metallophosphoesterase domain containing 2 (MPPED2)^50^.

Contrastingly, a larger number of targets associated with inhibition of apoptosis or stimulation of cellular proliferation were found in transitional carriers (Table 2). Overexpressed genes that were associated with tumor progression (proliferation factors) within this animal cohort included nephroblastoma overexpressed gene (NOV or CCN3), which has been associated with promotion of cell survival and attachment^51,52^. Similarly, protein phosphatase 1, regulatory (inhibitor) subunit 16 A (PPR1R16A) has been identified as a molecular marker of endometrial carcinoma^53^ as well as an inhibitor of apoptosis in thymocytes^54^. Homebox D9 (HOXD9) is an oncogene that promotes cellular invasion^55^, and T-box 3 (TBX3) is thought to contribute directly to tumor formation^56^. Amongst overexpressed genes associated with anti-apoptotic pathways were SRY-box 18 (SOX18)^57^ and BCL2/adenovirus E1B 19 kDa interacting protein 3-like (BNIP3L)^58^.

### Persistent phase of infection, immune-regulatory

During the persistent phase of infection, a large number of immune-regulatory genes that were overexpressed within the persistently infected carriers were associated with anti-inflammatory activation, prostaglandin synthesis or promotion of Th2-associated pathways (Table 3; Fig. 2b). These included two distinct variants of phospholipase A_2_ (PLA2G2A)^59^ as well as MAP3 kinase 8 (MAP3K8, also known as Tumor progression locus 2; Tpl2)^60^. Phospholipase A_2_ is an important component of the arachidonic acid cascade, and promotes downstream production of prostaglandin E_2_ (PGE_2_). PGE_2_ is an important regulator of immune responses during chronic infection, and has a critical function in suppressing Th1-associated cytotoxicity while promoting a Th2-polarized humoral response^61^. Additional immune-regulatory cytokines that were overexpressed in FMDV carriers included IL-33, which has been associated with expansion of Treg cells^62^, thereby also contributing to suppression of cytotoxicity. Additionally, several targets of this functional category that were overexpressed in FMDV carriers have been associated with direct inhibition of anti-viral responses, including secretory leukocyte peptidase inhibitor (SLP1)^63^, guanylate binding protein 4 (GBP4)^64^, and leukocyte associated immunoglobulin like receptor 1 (LAIR1; CD305)^65^. The multifunctional pro-inflammatory cytokine tumor necrosis factor-α (TNFα) was similarly overexpressed in FAE of the carrier cohort. This is consistent with a previous study that investigated regulation of inflammatory cytokines in the nasopharyngeal mucosa through different phases of FMDV infection^39^. Within the immune-regulatory genes, three of the targets that were overexpressed within the non-carrier cohort were associated with signaling and survival of macrophages (HCK, ELK4, CREB5; Table 3). However, there was less consistency of pathway associations of gene regulation in this group.

### Persistent phase of infection, apoptosis and proliferation

Among genes associated with regulation of cellular proliferation and apoptosis, multiple genes involved in anti-apoptotic pathways or the promotion of cellular division and proliferation were overexpressed in the FMDV carriers (Table 4; Fig. 2b). Among the genes with the highest overexpression in FMDV carriers were serine/arginine-rich splicing factor 9 (SRSF 9), high mobility group nucleosome binding domain 5 (HMGN5), insulin-like growth factor 2 mRNA binding protein 3 (IGF2BP3), zinc finger protein 280B (ZNF280B), hairy/enhancer-of-split related with YRPW motif-like (HEYL), and YKT6 v-SNARE homolog (YKT6), which have all been associated with tumor progression and the inhibition of pathways that promote apoptosis and regulation of cellular growth^66–71^. Contrastingly, genes within this functional category that were overexpressed in non-carriers were generally promoters of apoptosis or inhibitors of cell growth (Table 4 Fig. 2a). These included well characterized tumor suppressors such as sal-like 2 protein (SALL2) and testis specific gene antigen10 (TSGA10)^72,73^. Other targets, including proline-rich AKT1 substrate 1 (AKT1S1), and cyclin-dependent kinase 2-interacting protein (CINP) have been ascribed functions associated with controlling cell growth by promotion of apoptosis^74,75^

### Characterization of the cellular immune response by immunomicroscopy

In order to further characterize the host response to FMDV, the local cellular immune response at mucosal infection sites during the transitional phase of FMDV infection was investigated by immunomicroscopy. This was achieved by phenotypic determination of T cell populations within replicate cryosections from the same nasopharyngeal tissue samples that were used for laser-capture microdissection and transcriptome analysis. The analysis was performed on distinct regions of FAE, which is the known micro-anatomic site of persistent FMDV infection. The FAE has several distinct structural and functional characteristics including attenuation (thinner depth) relative to adjacent non-lymphoid epithelium, close proximity to subepithelial MALT follicles, an indistinct basal architecture, and a heterogeneous population of embedded non-epithelial cells (leukocytes)^11^.

**Figure 4:**
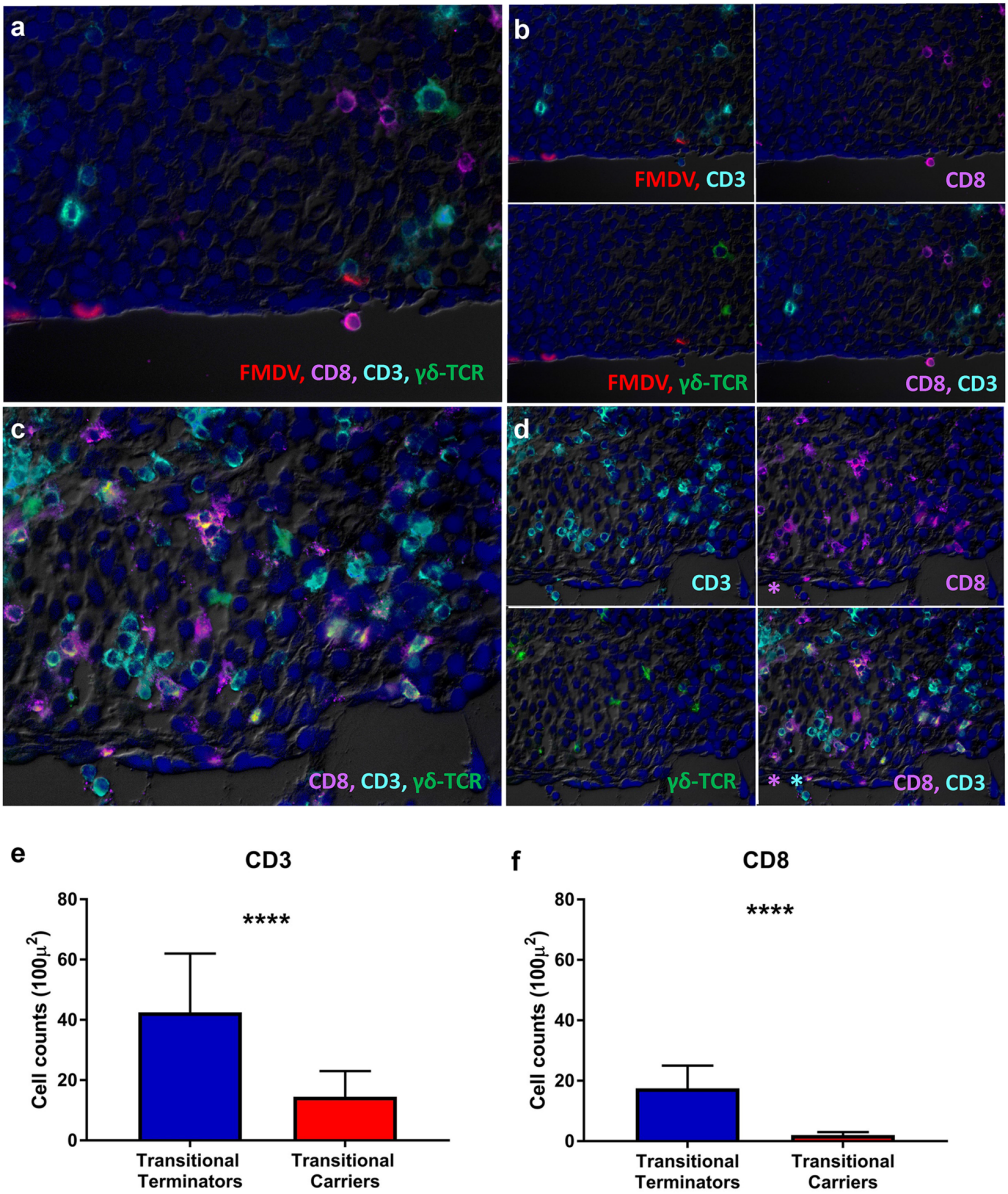
Variable T cell populations in bovine nasopharyngeal mucosa during the transitional phase of infection. Immunomicroscopic imaging of nasopharyngeal mucosa from transitional carrier (**a,b**) and transitional terminator (**c,d**). (**a**) FMDV structural antigen (red) is localized to scarce epithelial cells within the FAE of a transitional carrier. Few lymphocytes expressing variable combinations of CD3 (aqua), CD8 (purple) and γδ-TCR (green) are present within the subepithelial compartment (**b**) Selective channel combinations show that lymphocytes in close proximity of FMDV-infected cells are CD3+/CD8−/γδ-TC− (non-CTLs). (**c)** There is a marked abundance of lymphocytes expressing variable combinations of CD3 (aqua), CD8 (purple) and γδ-TCR (green) in epithelial and subepithelial compartments of the transitional terminator. (**d**) Selective channel combinations demonstrate variable co-localization patterns of phenotypic markers including CD3^+^/CD8^+^, CD3^+^/CD8^−^, CD3^−^/CD8^+^, CD3^+^/CD8^+^/γδ-TCR^+^ (**e,f**) Counts of CD3^+^ (**e**) and CD8^+^ (**f**) populations in serial sections demonstrated significantly greater numbers of CD3^+^ and CD8^+^ in the nasopharyngeal mucosa of transitional terminators compared to transitional carriers (p < 0.0001). Counts of cells expressing γδ-TCR were not significantly different between transitional terminators and transitional carriers. Bar charts show median counts with 95% confidence intervals.

Within the transitional carrier animals, FMDV structural antigen was localized to scarce epithelial cells within the FAE regions (Fig. 4a,b). There was no detection of FMDV antigen in transitional terminators (Fig. 4c,d). However, within the nasopharyngeal mucosa of transitional terminators, there was an abundance of small, round cells characterized as CD3^+^ , CD8^+^ , or CD3^+^/CD8^+^ (T lymphocytes). These cells were individualized or in small clusters within the epithelial and superficial subepithelial compartments in regions typically associated with persistent FMDV localization (Fig. 4c,d). Significantly fewer lymphocytes with these phenotypes were observed in the nasopharyngeal mucosa of transitional carriers (p < 0.0001 for both CD3 and CD8, Fig. 4e,f). The median count of CD3^+^ cells in 16 × 100 µm^2^ optical fields in transitional terminators was 43 (95% CI 19–62), and the corresponding count in transitional carriers was 15 (95% CI 9–23). Similarly, for CD8^+^ cells, the median count in transitional terminators was 18 (95% CI 9–25), and 2 (95% CI 0–3) in transitional carriers. Few cells expressing γδ-TCR were present in both transitional terminators and transitional carriers. There were more γδ-TCR^+^ cells present in transitional terminators (Fig. 4d), however, the difference was not significant.

## Discussion

Foot-and-mouth disease virus (FMDV) is capable of causing a persistent subclinical infection in the bovine nasopharynx. Although the existence of the FMDV carrier state has been thoroughly described, the immunological mechanisms involved are still poorly understood. Earlier works^76,77^ showed that persistent FMDV was localized to the bovine nasopharynx. More recent investigations^10,11^ have expanded upon that knowledge by demonstrating that within the nasopharynx, the FAE of the nasopharyngeal mucosa is the principal site of persistent infection. Additionally, concurrent detection of FMDV genome and capsid protein in subepithelial MALT follicles^11^ and regional lymph nodes^14,78^ has been reported. In this current investigation, animal samples dissected by laser-capture microdissection (LCM) were analyzed by whole transcriptome microarray to investigate the regulation of the local immune response with micro-anatomic specificity. The analyses were elaborated by considering four categories of animals: infected and non-infected in two time periods, transitional and persistent phases. Significant trends observed in the gene expression data were further corroborated by phenotypic characterization of T cell populations by immunomicroscopy.

In a previous investigation from our laboratory^11^, it was demonstrated that the FMDV carrier state divergence occurred earlier than previously acknowledged. This was accomplished through intensive monitoring of FMDV shedding in OPF through the post-acute and persistent phases of infection in a large cohort of cattle. Determination of the latest time point at which FMDV was recovered from OPF samples from animals that subsequently cleared infection led to definition of the transitional phase of FMDV infection. This distinct phase of FMDV pathogenesis was defined as the stage of infection that bridges the acute and persistent phases and thus corresponds to the temporal and functional window during which infectious FMDV is cleared from the nasopharynx of animals that do not develop persistent infection. In this current investigation, micro-dissected samples from the bovine nasopharynx were used to test the hypothesis that regulation of the local immune response during the transitional phase of infection would be a critical determinant for clearance versus persistence of FMDV.

The overarching trends of altered gene regulation amongst cohorts suggested that clearance of FMDV during the transitional phase of infection was associated with activation of a cell-mediated, cytotoxic response (Fig. 3). Additionally, multiple genes associated with pathways of apoptosis-induction or inhibition of cellular proliferation were upregulated in animals that had cleared infection (transitional terminators and non-carriers). Contrastingly, in animals that maintained persistent FMDV infection, there was an overarching pattern of activation of pathways associated with promotion of an antibody-mediated immune response as well as inhibition of apoptosis-associated pathways.

Microarray findings during the transitional phase of infection indicated that amongst genes associated with immune regulatory functions, numerous targets overexpressed in transitional terminators were directly associated with the activation or stimulation of cytotoxic T cells. This finding is consistent with the classical knowledge that activation of cell-mediated immunity is a critical mechanism of clearance of virus-infected cells^79^. The microarray data also indicated upregulation of two bovine major histocompatibility complex class I genes (BOLA; α-chain and non-classical MHC I) targets in FAE of transitional terminators, further supporting an activated cytotoxic response through the critical interaction of MHC I and CD8^+^ T cells. However, as a third variant of BOLA class I was simultaneously overexpressed in the transitional carriers, the direct functional relationship of these findings is not clear. Expression of NFκB was higher in FAE of transitional terminators, which had recently cleared FMDV infection, compared to the transitional carriers which were still infected. This finding is consistent with previous works that have found that the papain-like leader protease of FMDV is capable of degrading NFκB as a path of down-regulating the cellular antiviral response^80^.

In contrast to the pattern of activation of a cellular immune response in FAE of the transitional terminators, immune-regulatory genes that were overexpressed in the transitional carriers were more consistently associated with inhibition of T cell activation or induction of cellular senescence, while some targets suggested a Th2 polarization. A Th2 versus Th1 bias is associated with activation and subsequent expansion of CD4^+^ T helper cells. Upon initial activation by interaction of the TCR of immature CD4^+^ T cells with MHC II-bound peptides presented by antigen presenting cells (APCs), the T helper cells clonally expand and give rise to effector cells of one of three distinct phenotypes; Th1, Th2 or Th17. These functionally distinct T helper cell phenotypes differ in cytokine secretion patterns; while Th1 cells secrete IFN-γ and TNF-β, promoting protection against intracellular pathogens, the Th2 cells secrete IL-4, IL-5, IL-10, and IL-13, which promote antibody production by activated B cells^81^. The Th1 versus Th2 responses are mutually antagonistic, and promotion of either will suppress the other. Th2-polarization is associated with severe clinical disease and impaired virus clearance in respiratory syncytial virus (RSV) infection in children^82^, and has also been associated with the differential clinical outcomes and treatment responses in hepatitis C (HCV) infection^83,84^.

The transcriptomic finding of a differentially activated cell-mediated cytotoxic response during the transitional phase of infection was further corroborated by microscopic phenotypic characterization of T lymphocyte populations in replicate nasopharyngeal tissue samples. Immunomicroscopic characterization of tissue sections combined with systematic quantitation of distinct phenotypes demonstrated significantly greater numbers of CD3^+^ and CD8^+^ cells in the nasopharyngeal mucosa of transitional terminators compared to transitional carriers. The minimal detection of δγ-TCR-expressing cells, suggested that the critical T cells involved in these processes are αβ-TCR-expressing T cells. These bimodal findings strongly support the role of the cytotoxic T cell response in preventing the FMDV carrier state in these animals. Additionally, this finding is consistent with a previous publication which showed scarce numbers of T cells present in the proximity of FMDV-infected foci in persistently infected carriers^11^. In that study, it was similarly concluded that in carrier cattle, the majority of T lymphocytes detected adjacent to FMDV-infected epithelial cells were presumptive T-helper cells based on a CD3^+^/CD8^−^ phenotype^11^. This is consistent with the enhanced Th2 response in carriers suggested herein.

The persistent phase of FMDV infection was also characterized by distinct transcriptomic alterations. The carrier state is marked by continued presence of low level FMDV replication in the nasopharyngeal mucosa of the carrier animals. Contrastingly, the non-carrier cohort had cleared FMDV during the transitional phase of infection, which in the current study occurred approximately two weeks before the harvest of the persistent phase tissue samples.

The microarray analysis of samples from persistently infected carriers suggested an induced anti-inflammatory response including overexpression of multiple targets associated with prostaglandin E_2_ (PGE_2_) synthesis, as has been previously shown^21^. PGE_2_ can be synthesized by a variety of cell types through the action of phospholipase A upon cellular lipid membranes to initialize the arachidonic acid cascade^85^. PGE_2_ is of particular importance during chronic infections as it functions to downregulate acute inflammatory responses and thereby limits tissue damage. The downstream effects of PGE_2_ are broad and include direct inhibition of cytotoxicity and Th1 mediated cellular responses while driving a shift towards the less aggressive forms of Th2 and Th17 mediated responses^61^. Thus, induction of PGE_2_ synthesis as suggested by upregulation of phospholipase A_2_ is also suggestive of a Th2 polarization and promotion of an antibody-mediated immune response in persistently infected FMDV carriers. The pattern of regulation of immune-regulatory genes in the non-carrier cohort was less consistent. This is likely a reflection of virus having been cleared from these tissues approximately two weeks prior to harvest, and further emphasizes the importance of focusing on the transitional phase of infection in order to elucidate mechanisms of virus clearance.

Microarray analysis indicated a consistent pattern of regulation of genes associated with apoptotic- and cell proliferative pathways during the transitional and persistent phases. Specifically, genes associated with promotion of apoptotic pathways or inhibition of cell proliferation were generally overexpressed in the transitional terminators and non-carrier cohorts, whereas the opposite pattern was found in the persistently infected carriers and transitional carriers. It is striking that in the current investigation, a substantial subset of genes that were found to be overexpressed in nasopharyngeal tissues of FMDV carriers and transitional carriers were associated with tumor progression and cellular proliferation. Although these molecular pathways cannot be directly linked to persistence of FMDV, previous investigations have suggested an association between FMDV infection and an increase in cellular metabolism and extracellular matrix turn-over^86^. The same publication and a subsequent study also demonstrated an association between inhibition of apoptotic pathways and FMDV persistence^21,86^, which was supported by the results of the current investigation.

The combined findings of the current investigation suggest that an activated cytotoxic cellular response is a key function that is critical for clearance of FMDV-infected cells from the bovine nasopharynx. Contrastingly, promotion of a strong antibody-mediated response by Th2 polarization may inhibit cytotoxicity and thereby promote FMDV persistence. Both carriers and non-carriers mount a strong antibody response to acute FMDV infection, and it is not possible to distinguish FMDV carriers from non-carriers by routine serological methods^2,16,17,87^. Interestingly, although a strong humoral immune response elicited during the early stages of infection is essential for clearance of viremia and systemic infection, this antibody-mediated immunity is not sufficient to clear virus-infected cells during persistent infection. However, promotion of a strong Th2-mediated response may be a consequence of a strong survival pressure associated with clearing acute infection compared to lack of selective pressure to clear persistent infection. Thus, a Th2-biased immune response for the sake of clinical recovery may be prioritized over clearance of persistent infection, which does generally not impede the health of individual animals. Despite this, the transitional terminators and non-carriers represent animal cohorts that have succeeded in clearing both systemic (acute) and intra-cellular (persistent) virus, suggesting that an appropriate balance between antibody-mediated and cellular mechanisms is possible and likely essential to abrogate FMDV persistence.

Further investigations to elucidate immune mechanisms associated with the FMDV carrier state divergence should include characterization of FMDV-specific T cell responses during the transitional phase of infection. Additionally, quantitation of ratios of Th1- and Th2-associated cytokines in serum or secretions may further elucidate the role of this mechanism in prevention of the FMDV carrier state.

## Conclusions

The current work has demonstrated transcriptomic and immunophenotypic associations between induced cell-mediated immunity and promotion of apoptotic pathways with clearance of persistent FMDV infection. Contrastingly, promotion of a strong antibody-mediated response by Th2 polarization may inhibit cytotoxicity and promote FMDV persistence. Confirmation of these concepts will require further investigation of viral and host genomics and proteomics in larger numbers of animals. Further elucidation of such mechanisms may ultimately guide design of countermeasures that provide a more balanced immune response and thereby achieve prevention or cure of the FMDV carrier state, and potentially other persistent viral infections of veterinary and human relevance.

## Materials and Methods

### Animal experiments and definition of animal categories

This current study was based on a large scale experimental investigation of the FMDV carrier state divergence in cattle. Details of study design and disease progression has been previously published^11^. Animal experiments were carried out within BSL3-Ag facilities at Plum Island Animal Disease Center, New York. All procedures were carried out in accordance with guidelines specified within the associated experimental protocol (protocol 209-15-R), and were approved by the Plum Island Animal Disease Center Institutional Animal Care and Use Committee. The investigation presented herein was based upon analyses of tissue samples harvested from 13 FMDV-infected animals and 3 uninfected controls. In brief; cattle were infected with FMDV A_24_ Cruzeiro by intra-nasopharyngeal inoculation^88^, and were euthanized for tissue harvest at pre-determined time points after infection as previously described^11,17^.

Animals that were euthanized during the transitional phase of infection (8–21 dpi) were categorized as FMDV-positive or negative (“transitional carriers” or “transitional terminators”) based on isolation of FMDV from OPF and tissues at the time of euthanasia. By a similar approach, animals that were euthanized during persistent infection (post 28 dpc) were characterized as either FMDV carriers or non-carriers.

### Tissue samples

Necropsies with collection of up to 25 distinct tissue samples were performed immediately after euthanasia of experimental animals as previously described^11^. Samples for initial screening for FMDV genome and infectious virus by qRT-PCR and virus isolation (VI) consisted of tissue replicates of approximately 20 mg that were placed in individual tubes and frozen in liquid nitrogen vapor^89^. For each anatomically distinct sample, an adjacent specimen intended for LCM was embedded in optimal cutting temperature media (OCT; Sakura Finetek, CA) within a disposable embedding mold (Sakura Finetek, CA) and frozen above liquid nitrogen.

### Determination of FMDV carrier status

FMDV carrier status was determined based on detection of FMDV in OPF by virus isolation as previously described^11^. All animals that were classified as non-carriers had a minimum of 4 consecutive FMDV-negative probang samples on or later than 21 dpc whereas FMDV detection in OPF was consistent in the persistently infected carriers. The transitional phase animals were categorized based upon FMDV detection in OPF and/or tissues at the time of euthanasia. Thus, isolation of FMDV from either tissues or OPF lead to a status of transitional carrier, and FMDV-negative tissues and OPF lead to a status of transitional terminator.

### Laser-capture microdissection

Nasopharyngeal tissue samples from either the dorsal nasopharynx or dorsal soft palate, were selected for laser-capture microdissection (LCM) based upon detection of FMDV by qRT-PCR and VI. The LCM procedure was performed as previously described^11,18^ with minor modifications. In brief, distinct samples consisting of follicle-associated epithelium (FAE) were dissected from 10 µm cryosections from each selected tissue sample using an Arcturus XT™ LCM system. Dissected samples with a combined surface area of approximately 400 000 µm^2^ were captured onto individual CapSure Macro LCM caps (LCM0211, Life technologies) which were immediately mounted onto micro-tubes containing 50 µl of RNA extraction buffer (PicoPure™). RNA extraction was performed using the PicoPure™ RNA isolation kit (KIT0202, Life Technologies) with a final elution volume of 12 µl. FMDV RNA content in micro-dissected tissue samples was determined by qRT-PCR as previously described^11^. All micro-dissected FAE samples from transitional carriers and carriers were confirmed to contain FMDV RNA whereas samples from transitional terminators and non-carriers were FMDV-negative.

### Multi-channel immunomicroscopy

Cryosections from selected nasopharyngeal tissue samples from transitional terminators and transitional carriers were analyzed by multi-channel immunomicroscopy as previously described^11,90^. Slides were examined with a wide-field, epifluorescent microscope, and images were captured with a cooled, monochromatic digital camera. Images of individual detection channels were adjusted for contrast and brightness and merged in commercially available software (Adobe Photoshop CC 2017). Antibodies for phenotypic characterization of lymphocytes were rabbit monoclonal anti-human CD3 (confirmed cross-reactivity with bovine CD3, SP7 ab16669, Abcam), mouse mono-clonal anti bovine CD8 (MCA837G, AbD Serotec), and mouse mono-clonal anti-bovine γδ TCR (CACTB81A, Washington State University item number BOV2057). FMDV VP1 was detected using in-house derived mouse monoclonal antibody 6HC4^91^.

Quantitation of CD3^+^ and CD8^+^ lymphocyte populations was performed on two sections from both groups of animals. A grid of 100 × 100 µm^2^ squares was applied to 20x magnification images with grid placement adjusted so that at least 8 squares were placed within the natural boundary of the epithelial surface, covering the surface epithelium and adjacent subepithelial regions (total area: 200 µm depth × 400 µm width per section). The required definition to register a counted cell within each square of the grid was a visually identifiable nucleus and cytoplasm with clearly associated staining of either phenotypic marker within the cytoplasmic profile. Cell counts within 16 grid squares of each marker and animal category were compared using the non-parametric Mann Whitney test in Graphpad Prism 7.01 software.

### Bovine whole transcriptome micro array

The bovine whole genome expression microarray^86^ contains 45220 features, of which 43710 are 60-mer sense DNA probes based on non-redundant bovine mRNAs and expressed sequence tags (ESTs) from the NIH genetic sequence database (http://www.ncbi.nlm.nih.gov/genbank/). Glass slides with four 44 K high-density arrays to a slide were produced by a commercial supplier (SurePrint HD, G2514F; Agilent).

RNA was extracted with the PicoPure™ RNA isolation kit as described above. The quantity and quality of extracted RNA was assessed on an Agilent 2100 Bioanalyzer using the RNA 6000 Pico kit (Agilent, catalog number 5067–1513). The RNA concentrations in purified samples were between 1–2 ng/µl which was below the recommended threshold for generating valid RIN numbers, but within the applicable range for the whole transcriptome amplification kit. The extracted RNA was amplified by with the REPLI-g whole-transcriptome amplification single-cell kit (cat no. 150063, Qiagen) following the manufacturer’s instructions, with an input volume of 8 µl RNA. The REPLI-g kit was selected specifically due to manufacturer’s product description including documented low amplification bias. Additionally, oligo-dT primers were used to selectively enrich poly-A-tailed mRNA for amplification, matching the design of the bovine whole transcriptome microarray which intentionally has a low probe/poly-A tail distance. Amplified DNA was purified using Agencourt® AMPure® XP magnetic beads (cat. no. A63880, Beckman Coulter, Indianapolis, USA) and fragmented by heating to 95 °C for 30 minutes. The purified and heat-fragmented DNA was run on an agarose gel to confirm good fragmentation and size distribution. Fragmented DNA was labeled with cyanine (Cy) 3- and 5 using the SureTag DNA labeling kit (cat. no. 5190–3400, Agilent), purified with 30 K Amicon cartridges and hybridized to the microarray slides using the Hi-RPM Gene Expression Hybridization Kit (cat. no. 5190-0404, Agilent).

Cy3- and Cy5-labeled DNA from two tissue samples was hybridized to paired arrays in a dye-swap arrangement, for a total of four tissue samples per slide. The slide assemblies were incubated for 18 hours at 65 °C in a rotating oven set to 10 revolutions per minute. After the hybridization, array slides were washed following the manufacturer’s recommendations, coated with Cy5-stabilization and drying solution (cat. no. 5185–5979; Agilent) and scanned immediately with a GenePix 4000B scanner (Molecular Devices).

### Microarray data analysis

Background correction, normalization of the microarray data, and data analysis were performed as previously described^21^. The probes were not pre-filtered. Contrast matrices were set up for six comparisons, (i) between the temporally aligned paired groups of FMDV-infected animals (transitional carriers vs. transitional terminators and carriers vs. non-carriers), and ii) between each of the four animal categories and the uninfected controls. For each contrast and probe, log_2_ fold changes of signal intensity and p-values were calculated as previously described^92^. Unless stated otherwise, all fold change analyses are based on the log_2_ values (log_2_ fold change, log_2_FC). To account for multiple testing within a contrast, p-values were adjusted using the Benjamini and Hochberg^93^ method to control the false-discovery rate (FDR). Values adjusted with this method are bounds on the FDR and are referred to as q-values. A q-value of less than 0.05 was considered significant; accordingly, the expected proportion of false discoveries is controlled to be less than 5%^94^. Accordingly, probes with q-values (adjusted p-values) > 0.05 were removed from the output. Most of the subsequent analyses are based on the relative difference in signal intensity between transitional carriers and transitional terminators and between carriers and non-carriers. In these comparisons, probes that have higher signal intensity in transitional terminators or non-carriers have positive log_2_FC values; negative values indicate probes that had higher signal intensities in transitional carriers or carriers. Probes were ranked by log_2_FC, and only the top and bottom 0.1% of probes (i.e., the most strongly up- or downregulated probes) were further examined.

Within the filtered data sets, literature searches were used to separate identified targets into either of two functional categories; immune regulation or, apoptosis or cell proliferation.

### Data availability

The microarray data set generated within this study is available through the Gene Expression Omnibus data base^95^ with accession number GSE104058 (http://www.ncbi.nlm.nih.gov/geo/query/acc.cgi?acc = GSE104058).
